# Comprehensive analyses identify potential biomarkers for encephalitis in HIV infection

**DOI:** 10.1038/s41598-023-45922-6

**Published:** 2023-10-27

**Authors:** Shitao Wang, Xiangqian Ding, Zongyou Li, Feng Rao, Hui Xu, Jinghong Lu, Xuelu Ma, Mengen Zhang, Zhenrong Xie

**Affiliations:** 1 Department of Neurology, Affiliated Fuyang People’s Hospital of Anhui Medical University, Fuyang, 236000 China; 2 Department of Neurosurgery, Qilu Hospital of Shandong University, Jinan, 250012 China; 3 The Medical Biobank, First Affiliated Hospital of Kunming Medical University, Kunming, 650032 China

**Keywords:** Neuroscience, Neuroimmunology

## Abstract

Human immunodeficiency virus encephalitis (HIVE) is a severe neurological complication after HIV infection. Evidence shows that genetic factors play an important role in HIVE. The aim of the present study was to identify new potential therapeutic targets for HIVE. Differentially expressed gene (DEG), functional annotation and pathway, and protein–protein interaction analyses were performed to identify the hub genes associated with HIVE. Gene co-expression analysis was carried out to confirm the association between the hub genes and HIVE. Finally, the role of the hub genes in HIVE therapy was evaluated by conducting drug–gene interaction analysis. A total of 20 overlapping DEGs closely related to HIVE were identified. Functional annotation and pathway enrichment analysis indicated that the markedly enriched DEG terms included ion transport, type II interferon signaling, and synaptic signaling. Moreover, protein–protein interaction analysis revealed that 10 key HIVE-related genes were hub genes, including *SCN8A*, *CDK5R2*, *GRM5*, *SCN2B*, *IFI44L*, *STAT1*, *SLC17A7*, *ISG15*, *FGF12*, and *FGF13*. Furthermore, six hub genes were co-expressed with HIVE-associated host genes in human brain tissue. Finally, three hub genes (*STAT1*, *ISG15*, and *SCN2B*) interacted with several inflammation-associated drugs. These findings suggested that *SCN8A*, *CDK5R2*, *GRM5*, *SCN2B*, *IFI44L*, *STAT1*, *SLC17A7*, *ISG15*, *FGF12*, and *FGF13* may be new targets for diagnosis and therapy of HIVE.

## Introduction

There is growing evidence that the nervous system is often affected in human immunodeficiency virus (HIV) patients^1,2^. When HIV enters the central nervous system either as a cell-free virus or via CD4 T cells that infect perivascular macrophages, it causes a neuroinflammatory response that leads to HIV encephalitis (HIVE)^3–7^. Some promising HIVE biomarkers have been identified in previous studies. IL-16 is a biomarker for HIVE in human brain tissue^8^. Aquaporin-4 plays a role in the pathogenesis of HIV-associated neurocognitive disorders, including HIVE and cerebral cortex lesions^9^. Viral protein R is expressed in HIVE brain tissue samples, and further analysis found that it is present in the macrophages and neurons^10^. MAP2e has been identified as a biomarker of white matter lesions in HIVE^11^. P-glycoprotein also has an important role in the pathogenesis of brain disease in HIVE^12^.

Combination antiretroviral therapy has been demonstrated to be effective in inhibiting HIV-1 replication. However, the virus cannot be completely eradicated from the body^13^. Although HIV pathogenesis in the brain has been reported^14^, the pathogenic mechanisms of HIVE remain unclear. Therefore, it is increasingly important to elucidate the molecular mechanisms of HIVE and provide new strategies for its diagnosis and therapy.

To identify new potential therapeutic targets for HIVE, differentially expressed gene (DEG), functional annotation and pathway enrichment, protein–protein interaction (PPI), gene co-expression, and drug–gene interaction analyses were performed.

## Materials and methods

### DEG identification

HIVE was used as a keyword to search for HIVE patients in the GEO Datasets (https://www.ncbi.nlm.nih.gov/). Because the GSE3489 and GSE35864 series are datasets of frontal cortex brain tissues, both were included in the study analysis. DEGs were identified with the GSE3489 and GSE35864 series using GEO2R (http://www.ncbi.nlm.nih.gov/geo/geo2r). The GSE3489 series on the GPL8300 platform (Affymetrix Human Genome U95 Version 2 Array) and GSE35864 series on the GPL570 platform (Affymetrix Human Genome U133 Plus 2.0 Array). The GSE3489 included 16 HIVE and 12 HIV-seropositive without HIVE frontal cortex brain tissues, while the GSE35864 series included five HIVE and seven HIV-seropositive without HIVE frontal cortex brain tissues. Basic information for these two datasets were obtained from the National Coalition Building Institute (http://www.ncbi.nlm.nih). *P* value ≤ 0.05 and |logfold change| ≥ 1 were considered statistically significant. The Venn map was generated using the Venn tool (https://bioinfogp.cnb.csic.es/tools/venny/index.html). Overlapping DEGs were considered candidate genes for HIVE and were used for further analysis. Volcano plotting tool was used to generate volcano maps (http://soft.sangerbox.com/).

### Functional annotation and pathway enrichment analysis

Metascape is one of the most important bioinformatics knowledge databases. To identify whether the DEGs of interest were enriched in gene ontology categories or pathways associated with central nervous system inflammation, functional annotation and pathway enrichment analysis was conducted using the Metascape database (http://metascape.org/gp/index.html#/main/step1).

### PPI analysis

The primary role of the Cytoscape software is to assist in molecular and network analysis and to construct molecular interaction networks. To evaluate the interactions between proteins and to identify HIVE-associated hub genes, PPIs were first evaluated using the STRING database (https://string-db.org/cgi/input.pl), followed by construction of molecular interaction networks with Cytoscape software^15,16^.

### Gene co-expression analysis

Gene co-expression networks help to identify transcriptional regulatory relationships among candidate disease genes. HIVE is a brain disease and in order to evaluate the co-expression relationships between HIVE-associated hub and host genes in human brain tissue, gene co-expression analysis was carried out using the data from the brain tissue gene co-expression database^17^.

### Evaluation of potential role of hub genes in HIVE treatment

The Drug–gene Interaction Database provides user-friendly searching capability and filtering of information on drug–gene interactions and medicinal genome, integrating more than 30 trusted sources. To evaluate the potential role of hub genes in HIVE therapy, potential drug-interacting hub genes for HIVE therapy were explored using the Drug–gene Interaction Database^18^.

## Results

### DEG identification

**Figure 1:**
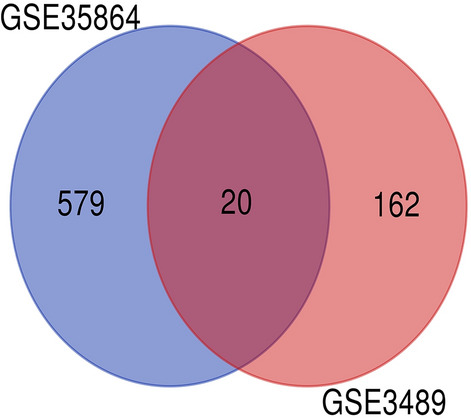
DEGs were identified from the GSE35864 and GSE3489 gene expression profiling datasets based on *P* value < 0.05 and **|**logfold change**| **≥ 1. The two datasets share 20 overlapping DEGs.

After analyzing the GSE35864 and GSE3489 series, 599 DEGs were identified in the GSE35864 series (Table 1) and 182 DEGs were identified in the GSE3489 series (Table 2). Further analysis showed that 20 genes overlapped in the two datasets (Table 3), where expression of five genes was upregulated and expression of 15 genes was downregulated in both datasets (Fig. 1). Of the 20 genes, two have been reported to be associated with HIVE (*OAS1* and *BTN3A2*)^19,20^.

**Table 1:** The 599 DEGs were identified in the GSE35864 series.

Series	DEGs
GSE35864	*SEC23A, RAB2A, HMGCR, RIT2, MAP4, ARHGAP20, TMEM130, INPP5F, CNTN3, SLC25A40, CHGB, SALL1, FAM3C, PRKAA2, RGMB, AMPH, C1QTNF4, MPPED2, TMEM178B, PAK1, PRKAR2B, PPP1R2, TOMM20, SYT1, SLC1A6, TUBA4A, LSM11, STYK1, LINC01128, NAP1L2, CITED2, RTN1, GPSM2, SMIM17, PRKAG2-AS1, TMX4, TMEM155, HCN1, FAM234B, EFNA5, NETO2, CFAP36, GDA, XK, SLC16A14, TUBB2A, PTK2B, GOT1, PCSK1, LINC00320, NRN1, ACTR3B, ACTL6B, EPDR1, CYP4X1, PARP12, ATP6V1B2, STXBP5L, RAB3C, CALB1, SHISA6, PLK2, PPP3CB, DNAJA2, CLCA4, SERPINF1, ST6GALNAC5, BST2, LYRM9, DHRS11, KIAA2022, STX1A, NGEF, NAP1L3, BMPR2, B3GALNT1, RBP4, BASP1, PRKCB, NTRK2, ADCYAP1, SP110, PNMA2, CRYM, ARPP21, PLCB1, HIVEP2, LAMB1, NUDT11, G3BP2, CPNE4, FOXO4, SLC8A1, GAREM2, KCNJ4, ANKH, INA, SYNGR3, GNG3, ELAVL4, SLC16A6, RFPL1S, KCNA1, PPP3R1, DMXL2, UCHL1, SCG2, DCAF6, PPP3CA, SERPINI1, SLC17A6, GNAS, NLK, EID2B, HTR2C, GUCY1A2, JADE3, ARHGEF9, NBEA, MDH1, TBC1D30, RAD23B, ZNF808, ENPP5, FAM19A2, CACNA1E, TESPA1, RTF1, FBXO9, MAGEE1, NEFH, SLITRK5, RAPH1, CHML, TIAM2, CHI3L2, ZNF385B, CABP1, NECAP1, VAMP1, FGF9, CREG2, KIFAP3, ATP6V1H, SYT13, SLC1A1, FREM3, HPCAL1, PI4KA, SRD5A1, MYT1L, C11orf87, GRIN1, PTGR1, NAP1L5, ATAD1, CLEC2L, RARRES3, ZNF25, SCN2A, DTX3L, LRP8, TAGLN3, RBFOX1, ENSA, CLSTN3, PCDH7, GBP1, CAMTA1, OPCML, MRAP2, AKAP12, ITPKB, FAM102B, FXYD7, CPLX1, CDS1, CACNG3, RAB3A, TUSC3, FAXC, CA10, C2orf80, ST8SIA3, PRPS2, DCLK1, NEFM, GPI, CDR1, STMN3, LDB2, SYN1, MICAL2, ADAM23, PVALB, NCDN, NRGN, CDC42EP3, PTPN5, LPCAT4, STAT4, HPRT1, FAM216A, CHST6, PNMAL1, VSNL1, PWAR6, SLITRK4, MAPK10, LRRC40, LRRC7, LINC00889, PDP1, ASNS, MYOT, KCNJ3, SYNE1, HIST1H2AC, PCDH8, TSPYL1, ANKRD29, OPRK1, GPR22, CHRM1, NCEH1, ARMC8, LOC101929748, CNTNAP2, EPHA4, BTN3A3, RYR2, BEX1, SCAMP1, KCNQ5, KRT222, LY6H, OLFM1, CAMK4, CBLN4, CPLX2, SNX10, KCNIP4, DYNC1I1, EGR1, LONRF2, CACNB2, TRIM37, TOX2, FBXW7, SERTM1, RBFOX2, KIAA1549L, CNR1, VSTM2A, FAR2, SLC12A5, RSPO2, ATP6V1C1, PPP2CA, LRP12, GABRA4, NRIP3, SSTR1, ATP1A1, NMNAT2, SORBS2, UBE2N, IFIT2, PCLO, GUCY1B3, YWHAZ, PGM2L1, PAIP2B, GPRASP1, LMO4, SYBU, SCN1A, ZNF204P, CYP26B1, PKIA, SCG3, IDS, SEC62, IFI16, SYNPR, FAM84A, PEG3, GABRB3, MS4A7, CXCL10, GNB5, PRKAR1B, SV2B, ITFG1, GPR17, HMGCLL1, HERC5, MFSD4A, AKAP5, GBP3, GLS, FBXL16, STS, CAP2, NPTX2, WDR17, ZFPM2, CCDC113, PIEZO2, SLC9A7, YWHAH, ROBO2, GPR158, ATP6V1G2, RAB27B, SNAP25, RSAD2, SHANK2, CAMK2B, CDH18, CHL1, KLC1, SUB1, GLRB, CADPS2, PDK1, NCALD, WWTR1, UNC80, LRRC2, KIF3A, SLC39A10, KCNV1, ATP2B2, EID2, MEST, MAPRE3, SPIN3, RAB11A, SLC1A2, LOC100507557, COL5A2, CUL2, UNC5D, MPC2, CADPS, RASA1, GABRG2, LGALS3BP, DLGAP1, IRF7, GDAP1, TMEM35A, ARPC5L, SAMD9L, ACSL6, EXOC8, NDRG4, NAPB, UNC13C, LDHA, HIPK2, RGS7, GABRA5, PCP4, HMP19, SGTB, NRXN3, NPTX1, RIMS2, HS6ST2, STXBP1, CIRBP, SVOP, PREPL, LOC440934, MRPS25, HOOK1, XAF1, GRIA1, FAM19A1, PKI55, STXBP5, FZD3, KALRN, TUBB4B, CAMKK2, HS6ST3, ENO2, ITPR1, SLC25A12, EIF5A2, EIF5A, NEGR1, FAM49A, SSX2IP, KSR2, NEFL, COX7B, ENC1, MAP2K1, ATP6V1A, KYAT3, OXR1, DGKB, PAK3, TRIM56, ATRNL1, REPS2, GBP2, CDH12, EPHX4, LOC100506563, CDC42, VWF, CHRM3, GABBR2, CDKL5, KCND2, HIST1H2BD, GAD2, INPP4A, LY86-AS1, ATP8A2, TSNAX, LINC00507, GABARAPL1, BCL11A, TAC1, HMGCS1, SMIM10L2A, HPCAL4, SST, RUNDC3B, DIRAS2, WBSCR17, HAR1A, NDFIP2, LINC01616, UBE2V2, MAP7D2, ADAM22, GRIA4, KCNAB1, FAM155A, RBM3, CNIH3, MAP4K4, CALY, HTR2A, SNCA, VDAC1, KCNJ10, ARHGAP26, GAP43, ERC2, PRICKLE1, GNG2, SLIT2, C3orf80, TRHDE, TMED2, MKRN1, CCK, MLLT11, DRP2, ERICH3, PSMB8, OLFM3, FKBP1B, ABRACL, TUBB3, WNK1, CHN1, AHNAK2, RNF175, NRSN1, PPM1E, DLX1, LPAR1, PDK3, DNAJB14, GRIN2A, RASGRF2, FMNL2, LINC00403, LOC285147, QPCT, LHPP, NRXN1, STMN2, GRIA3, CTXN3, IRF9, ATP2A2, RGS7BP, PARP9, RXFP1, TBC1D9, JAZF1, SLC26A4-AS1, NELL2, SHC3, NXPH1, NIPAL2, PIANP, DPY19L2P2, PCSK2, GAD1, SCG5, RNF128, CTNNA3, AP3B2, SYT16, NDFIP1, SRSF3, AAK1, GNA13, EGR3, SNAP91, PPP4R4, CBLN2, CNKSR2, GPM6A, CCL2, PSMA5, ZC2HC1A, LNX1, SYT4, ADD2, GULP1, MAL2, ACSL4, NDRG3, SATB1, FGF14, ELOVL4, BEX5, NDUFA5, CSRNP3, GABRA1, GRIA2, GLS2, REEP1, ATP2B1, LMBR1, DPP6, PARM1, GPR26, RPH3A, MAP3K9, NXPH2, BRWD1, FAM49B, ADCY1, UBE2QL1, NWD2, RAB39B, BCAS4, SNCB, IFIT3, ARL6, HECW1, CACNA2D3, MCTP1, HOPX, MEF2C, LAMP5, YWHAB, TRAPPC6B, TRAPPC13, SYP, SCN3B, SYN2, PGK1, AFF3, COPG2IT1, EEF1A2, LINC00844, CDH13, BTN3A2, IFI44L, CNTN1, GRM5, RGS4, CDK5R1, HLF, PWAR5, OAS1, FGF13, B4GALT6, SCN8A, FGF12, NSG1, CDK5R2, SLC17A7, SCN2B, STAT1, ISG15*

*P* value ≤ 0.05 and |logfold change| ≥ 1 were considered statistically significant.

**Table 2:** The 182 DEGs were identified in the GSE3489 series.

Series	DEGs
GSE3489	*PRPF40A, PANX1, VAC14, NFE2, TAF4B, CD2BP2, KMT2D, PROSC, DUSP7, TRIM22, GJA1, MAPKAPK2, ABL2, EAPP, PCGF3, SH3BP2, PKD2, GRPEL1, COL1A1, TAF13, SLC6A1, PTBP3, NAPG, PIK3CG, APOBEC1, CCDC57, MAPKAPK3, BRMS1, RASGRF1, GLMN, FAS, DDX3Y, PTPN21, IFIT1, HCG4, ENOSF1, PKLR, TNFRSF11B, IL24, STAT5B, XIST, ENPEP, PRKG2, ATF2, TRPM1, HK2, EVI2B, MYBPC1, CELF1, IGLC1, PDE5A, MAPK8, TBX2, COL14A1, SPTBN1, ATP8A1, E2F1, HAL, CADM3-AS1, GPR171, FER, RASGRP3, BICC1, NEBL, PLA2R1, MAP2, ATP2B4, RB1, FXN, CYP7A1, PMS2P5, PF4V1, GDI2, DLX4, KNG1, CLCN3, BCAP29, NR2E1, PIK3R3, SPINK5, PTPRO, IL17A, ACTB, KCNC4, CSTA, UBL4A, FECH, ATP6AP2, LINC00893, KAZALD1, MAP1B, TNFRSF4, NOX1, EIF2S3, RAB40C, MX1, AMELX, ADH1B, KCND3, TRAF3IP3, LSR, GORASP2, FGFR2, ELAVL1, NCOA2, GJC1, EPHA7, UGP2, RAB5A, TERF2, NOP14-AS1, GPR176, ADAM10, MASP1, DLGAP2, NCK2, RUNX1T1, IGFBP2, BMP2, NPY1R, GCNT2, LCN2, TOP2B, WWP2, TSPAN4, TNFSF9, CYB5A, GABPB1, WSB1, VEGFA, AREG, GNA12, ZNF391, KIAA0101, IFITM1, CLEC10A, ETV1, RPS4Y1, ADAM11, CCNT2, OIP5, GPR18, ROR1, CSNK1A1, CALM1, SLC4A4, MAGI1, AIF1, PHTF2, AZU1, SLC11A1, LY96, ADCYAP1R1, NUDT21, HGF, H1F0, RHO, SMAP1, LSM7, TULP2, SPOCK1, ZNF165, CDH13, BTN3A2, IFI44L, CNTN1, GRM5, RGS4, CDK5R1, HLF, PWAR5, OAS1, FGF13, B4GALT6, SCN8A, FGF12, NSG1, CDK5R2, SLC17A7, SCN2B, STAT1, ISG15*

*P* value ≤ 0.05 and |logfold change| ≥ 1 were considered statistically significant.

**Table 3:** The 20 overlapping DEGs were identified in the GSE35864 and GSE3489 series.

Series	Overlapping DEGs
GSE3489, GSE35864	*CDH13, BTN3A2, IFI44L, CNTN1, GRM5, RGS4, CDK5R1, HLF, PWAR5, OAS1, FGF13, B4GALT6, SCN8A, FGF12, NSG1, CDK5R2, SLC17A7, SCN2B, STAT1, ISG15*

*P* value ≤ 0.05 and |logfold change| ≥ 1 were considered statistically significant.

### Functional annotation and pathway enrichment analysis

**Figure 2:**
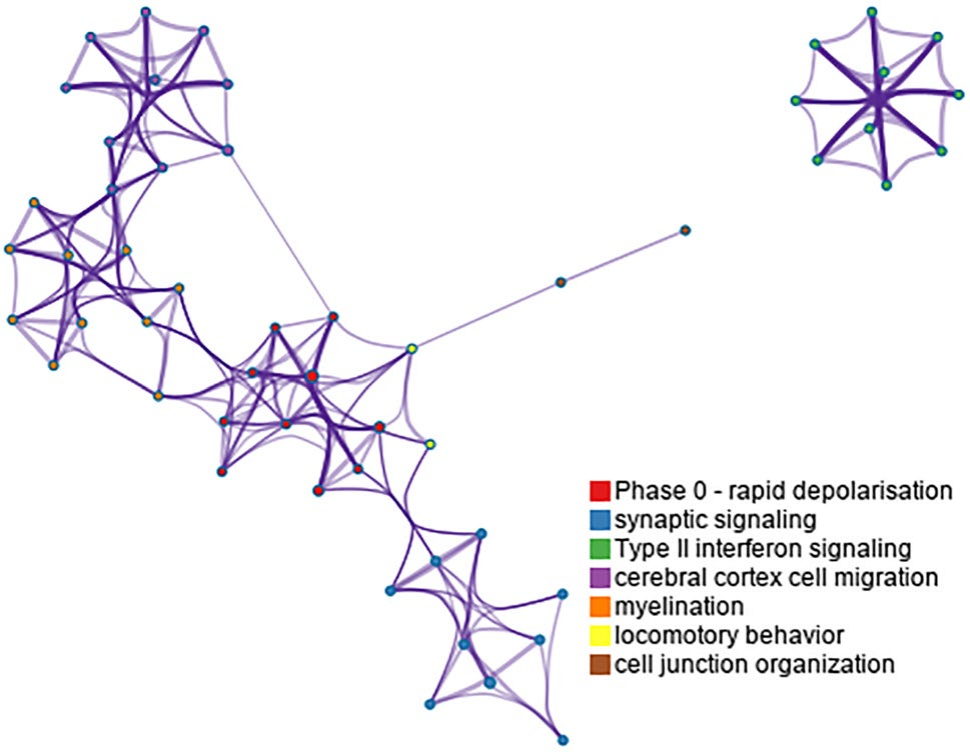
Functional annotation and pathway enrichment analysis of DEGs in the GSE35864 and GSE3489 gene expression profiling datasets. Network of enriched terms colored by cluster ID, where nodes that share the same cluster ID are typically close to each other.

The functional annotation and pathway enrichment analysis results showed that markedly enriched DEG terms included ion transport, type II interferon signaling, and synaptic signaling (Fig. 2).

### PPI analysis

**Figure 3:**
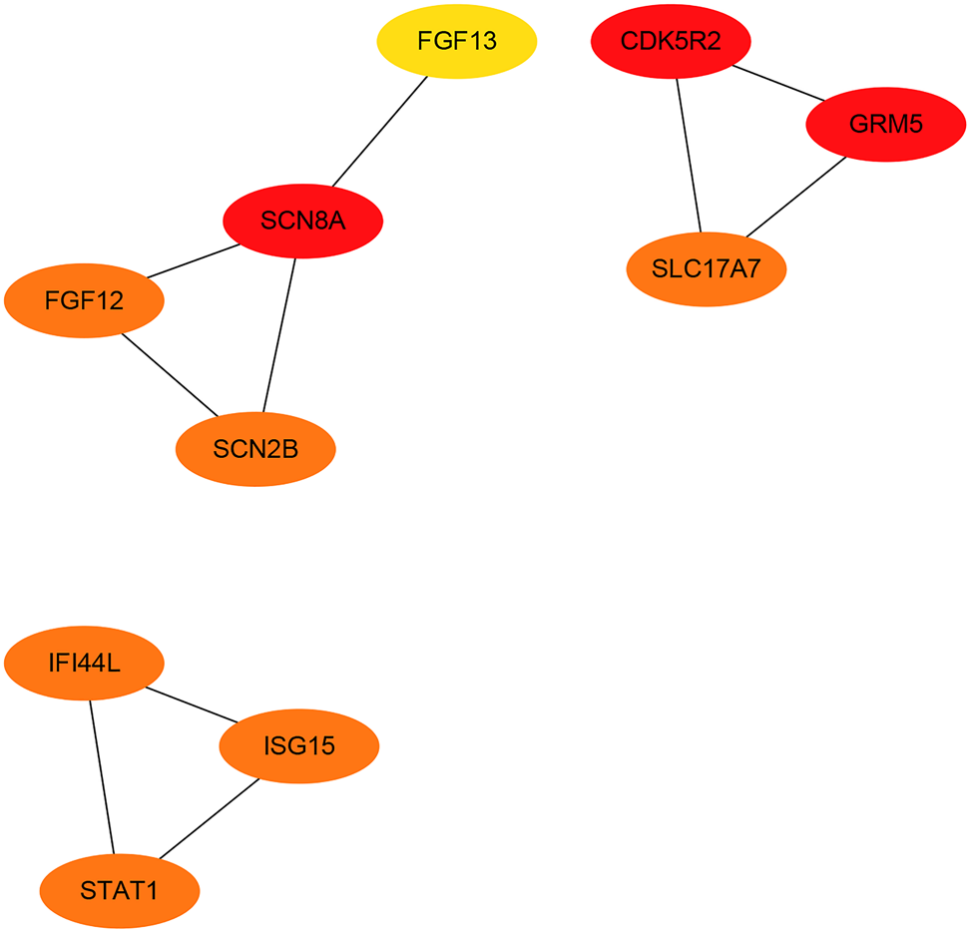
PPI network of the top 10 DEGs. The top 10 nodes in the network were ranked by degree method and were considered to be hub genes.

**Figure 4:**
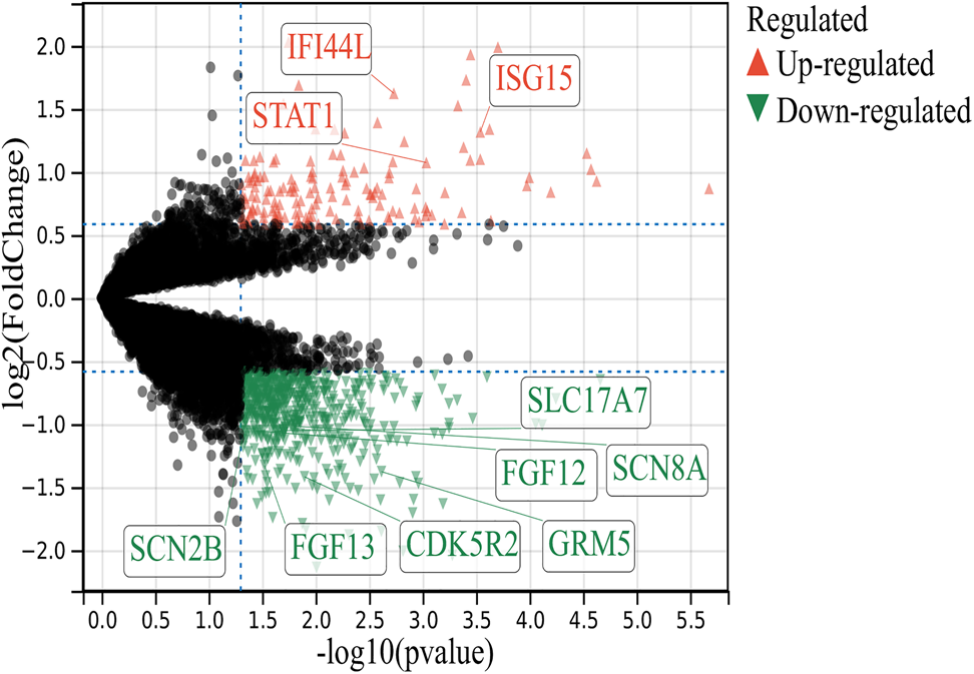
Locations of hub genes on the volcano plot illustrating DEGs between HIVE and controls after analyzing the GSE3489 gene expression profiling dataset.

Among the overlapping DEGs (excluding *OAS1* and *BTN3A2*), the top 10 nodes in the network were ranked by degree method (Fig. 3) and were considered to be hub genes. The hub nodes, including *SCN8A*, *CDK5R2*, *GRM5*, *SCN2B*, *IFI44L*, *STAT1*, *SLC17A7*, *ISG15*, *FGF12*, and *FGF13*, were considered to be hub genes associated with HIVE. Seven hub genes were downregulated and three were upregulated in human brain tissue (Fig. 4).

### Gene co-expression analysis

**Figure 5:**
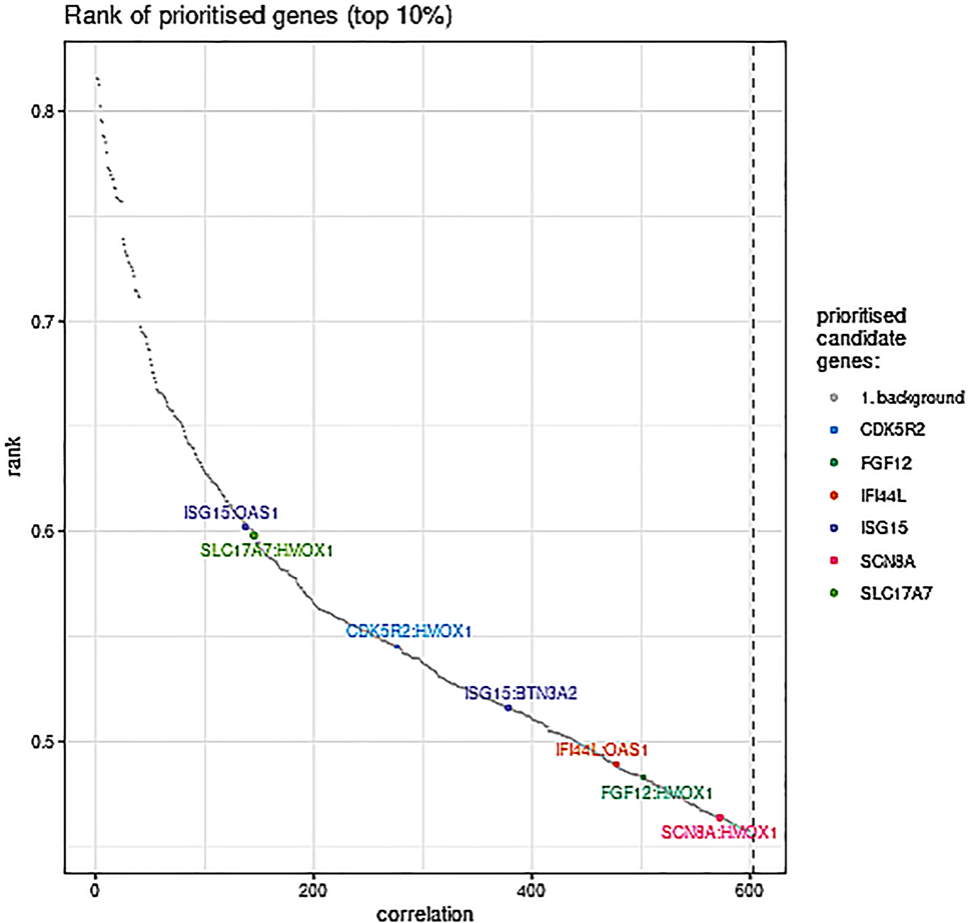
Analysis of gene co-expression in human brain tissue. Co-expression relationships were evaluated using data from the brain tissue gene co-expression database.

*OAS1*, *BTN3A2*, and *HMOX1* have been previously reported to be involved in HIVE^19–21^. *ISG15* was co-expressed with *OAS1* and *BTN3A2*, *SLC17A7* was co-expressed with *HMOX1*, *CDK5R2* was co-expressed with *HMOX1*, *IFI44L* was co-expressed with *OAS1*, *FGF12* was co-expressed with *HMOX1*, and *SCN8A* was co-expressed with *HMOX1* in human brain tissue (Fig. 5, Table 4).

**Table 4:** Top 10% of all known candidate gene correlations.

Gene1	Gene2	Correlation	Rank
*ISG15*	*OAS1*	0.60	134
*SLC17A7*	*HMOX1*	− 0.60	142
*CDK5R2*	*HMOX1*	− 0.55	265
*ISG15*	*BTN3A2*	0.52	351
*IFI44L*	*OAS1*	0.49	429
*FGF12*	*HMOX1*	− 0.48	469
*SCN8A*	*HMOX1*	− 0.46	552

### Evaluating the potential role of hub genes in HIVE treatment

After screening the Drug–gene Interaction Database^18^, several drugs that interacted with hub genes and linked to inflammation were discovered. For example, it was determined that *STAT1* interacted with garcinol and ipriflavone, *ISG15* interacted with irinotecan, and *SCN2B* interacted with zonisamide.

## Discussion

Although more and more HIVE biomarkers have been identified, the genetic basis of HIVE remains largely unknown. Previous studies have indicated that several genes were regulated in HIVE^22^. However, most of HIVE-associated genes still need to be uncovered. To further identify potential HIVE genes, the sample size in the present study was increased by combining data from different studies. Second, because there were some differences in gene expression in different brain regions, gene expression data for the same brain region (frontal cortex) were chosen for analysis.

Compared to the controls, 20 DEGs were successfully identified in HIVE frontal cortex brain tissue samples, two of which have been reported to be associated with HIVE risk. To determine whether these DEGs were enriched in gene ontology categories or pathways associated with central nervous system inflammation, functional annotation and pathway enrichment analysis was carried out using the Metascape database. The results showed that markedly enriched terms included ion transport, type II interferon signaling, and synaptic signaling. Interestingly, type II interferon signaling and ion transport have been reported to be associated with central nervous system inflammation in previous studies^23,24^.

In addition, PPI analysis was performed to determine the hub genes associated with HIVE. As a result, 10 new HIVE-associated genes were identified in the present study, including *SCN8A*, *CDK5R2*, *GRM5*, *SCN2B*, *IFI44L*, *STAT1*, *SLC17A7*, *ISG15*, *FGF12*, and *FGF13*. *Scn8a*^*dmu/*+^ mice showed reduced inflammation in response to lipopolysaccharide stimulation. Another study showed that the mast cells in *Scn8a*^*dmu/*+^ mice produced lower levels of IL-6 when confronted with the same stimulus^25^. In addition, it was shown that *FGF12* induced IL-6 and CXCL8^26^, that *GRM5* was a potential target for inflammatory disease^27^, and that *IFI44L*, *STAT1*, *ISG15*, and *FGF13* were biomarkers for inflammatory diseases^28–34^. Therefore, several hub genes were determined to be directly involved in immune response.

The construction of gene co-expression networks will help to identify transcriptional regulatory relationships among candidate disease genes. In the present study, several hub genes and HIVE-associated host genes were co-expressed in human brain tissue, where correlation coefficient greater than 0 indicated positive correlation (Table 4), while less than 0 indicated negative correlation*.* These findings can help identify potential functional relationships between disease candidate genes. The HIVE-associated host genes included *OAS1*, *BTN3A2*, and *HMOX1.* The expression level of *OAS1* was significantly upregulated in simian immunodeficiency virus-infected hippocampus compared to healthy controls. Furthermore, similar results were obtained analyzing the brain sections of HIVE patients^19^. The protein encoded by *BTN3A2* was shown to be associated with the adaptive immune response. The innate immune system is one of its related pathways. *BTN3A2* was upregulated in HIV-associated neurocognitive disorders in HIVE patients compared to HIV-associated neurocognitive disorder patients without HIVE^20^. Downregulation of *HMOX1* led to the central nervous system immune activation and neurocognitive dysfunction. Furthermore, shorter *HMOX1* (GT)n alleles decreased neuroimmune activation and thus significantly reduced the risk of HIVE^21^. Therefore, it can be speculated that there may be special regulatory relationships between hub genes and HIVE-associated host genes in human brain tissue. These findings further validated the role of newly discovered hub genes in HIVE.

Exploring the interaction between drugs and genes is conducive to linking the candidate genes in diseases to drug therapy, and, therefore, to further transformation of clinical application. In the present study, *STAT1*, *ISG15*, and *SCN2B* were found to interact with garcinol and ipriflavone, as well as irinotecan and zonisamide, respectively. Interestingly, recent studies have shown that garcinol and ipriflavone have anti-inflammatory effects, especially in the inflammation of the brain^35–38^, while irinotecan and zonisamide can induce inflammation^39–42^. Therefore, *STAT1*, *ISG15*, and *SCN2B* may be new targets for HIVE drug therapy. However, further research is needed to confirm these findings.

Several potential HIVE-associated genes were identified using integrative analysis in the present study. However, some study limitations need to be acknowledged. First, the study results were not further validated by functional assays. Second, the genetic dataset was relatively small and additional studies with a larger sample size and functional assays are needed to verify these results.

## Conclusions

In conclusion, *SCN8A*, *CDK5R2*, *GRM5*, *SCN2B*, *IFI44L*, *STAT1*, *SLC17A7*, *ISG15*, *FGF12*, and *FGF13* may be involved in the diagnosis of HIVE, among which *STAT1*, *ISG15*, and *SCN2B* are potential therapeutic targets for HIVE. These results are beneficial for translating gene research findings into clinical treatment, as they reveal the association between HIVE-related genes and inflammation-associated drugs.

## Data Availability

All the data we used in our study are publicly accessible at NCBI GEO database (Accession Number: GSE3489 and GSE35864; https://www.ncbi.nlm.nih.gov/geo/).

## Abbreviations

HIV: Human immunodeficiency virus
HIVE: Human immunodeficiency virus encephalitis
DEG: Diferentially expressed gene
*SCN8A*: Sodium voltage-gated channel alpha subunit 8
*CDK5R2*: Cyclin dependent kinase 5 regulatory subunit 2
*GRM5*: Glutamate metabotropic receptor 5
*SCN2B*: Sodium voltage-gated channel beta subunit 2
*IFI44L*: Interferon induced protein 44 like
*STAT1*: Signal transducer and activator of transcription 1
*SLC17A7*: Solute carrier family 17 member 7
*FGF12*: Fibroblast growth factor 12
*FGF13*: Fibroblast growth factor 13
DEGs: Diferentially expressed genes
PPI: Protein–protein interaction
